# Supplementary material for: Are carbon emissions trading and green financial instruments synergistic? -Comprehensive quantitative research based on content analysis
Source: PLoS One. 2024 Mar 7;19(3):e0298601. doi: 10.1371/journal.pone.0298601 (PMC10919601; doi:10.1371/journal.pone.0298601)
Supplement: S1 Data — (ZIP) [file pone.0298601.s001.zip › Data/地级市Z_F工作报告.zip!/╔┬╬≈/░▓┐╡╩╨/└┤╫╘╣1⁄2╓┌║┼í╛┬φ┐╦╩2╛▌═°í┐_2009.docx]

各位代表：

现在，我代表市人民政府向大会作工作报告，请予审议。并请政协委员和其他列席人员提出意见。

一、过去一年政府工作的回顾

2008年是不寻常、不平凡的一年。我们在中共安康市委的正确领导下，在市人大依法监督、市政协民主监督和大力支持下，坚持以科学发展观为指导，依靠全市人民的智慧和力量，经受了雨雪冰冻、地震灾害和金融危机的考验，全面完成了市二届人大四次会议确定的发展任务，在突破发展的征程上迈出了更加坚实的步伐。

经济平稳较快增长。全年完成生产总值233.7亿元，增长15.1%；全社会固定资产投资201亿元，增长35.8%，其中地方固定资产投资151亿元，增长52.3%；财政总收入26亿元，增长30%，其中地方一般预算收入7.49亿元，增长28.8%，一般预算支出57.2亿元，增长63.9%；社会消费品零售总额78亿元，增长24.9%；城镇居民人均可支配收入10150元，净增2099元，增长26.1%；农民人均纯收入2770元，净增514元，增长22.8%；物价涨幅5.1%，单位能耗下降4.5%。

项目建设成效显著。全市新开工项目1011个，增长60.2%。以交通为重点的基础设施项目扎实推进。襄渝铁路复线建设进展顺利，西康高速、川陕界高速安康段加快建设，十天高速安康段全线开工，安康机场迁建工作全面展开，县县通高速列入全省建设规划，地方道路建设加快实施。以工业为重点的产业类项目滞后状况明显改变。产业类项目投资62.84亿元，增长82.6%，占全社会投资总额的1/3，占地方投资总额的2/5，其中工业项目完成投资49.32亿元，增长90.6%。招商引资到位24.7亿元，引进外资3025万美元，外贸进出口总值达1000万美元。与省内外一批大企业集团的项目合作取得实质性进展。以生态建设为重点的环境保护力度加大。汉江中上游水土保持项目扎实推进，瀛湖核心区绿化任务按期完成，城镇污水垃圾处理项目按规划加快实施，全年完成造林绿化48.1万亩，治理水土流失940平方公里。以民生为重点的社会事业项目建设取得实效。列入地震灾区恢复重建的项目加快建设。

新型工业加快发展。按照“统筹城乡、循环经济、产业集群、项目支撑”的要求，组织编制了以月河川道石泉、汉阴、汉滨为主体，旬阳、平利为两翼的“一体两翼”区域发展规划和新型材料工业规划。省政府已将月河川道列为统筹城乡一体化发展综合实验区。安康工业园区在调整中加快建设，汉阴、石泉、紫阳、旬阳4个县域工业园区通过省上审定。特色品牌建设和名牌产品培育取得成效。全市实现工业产值135亿元，增长23%，新增规模企业28户。尧柏水泥、新型管材、双稀沃氏、天新捻丝等40个工业项目投产运营；旬阳电站、小金河大型铁矿、纳米氧化锌迁建扩能、南江河水电梯级开发、汉滨五氧化二钒等重点项目开工建设。列入当年开工、续建、竣工、前期的“四个十”工业项目推进计划如期实现。

三农工作继续加强。以推进土地流转和林权制度改革为突破口，积极创新一县一业、一村一品、强村大户、龙头牵引、集约经营的机制和组织形式，大力推广先进实用技术，加快农业发展方式转变。编制了规模、特色、效益农业发展规划。全年粮食总产82万吨、油料总产9.5万吨，分别增长9.2%和19.2%；猪羊禽饲养量365.5万头、135.2万只和1482.5万羽；产茧1.78万吨，产茶5800吨；烤烟、魔芋、蔬菜、中药材等大宗农产品产量大幅增长。劳务输出55.5万人，劳务收入49.5亿元。基础设施、村镇民居、主导产业、社会事业建设进一步加强，新农村示范村建设发挥了典型引路作用，农业综合开发和重点村的扶贫开发取得明显实效，特困村的经济社会发生了显著变化。县域经济发展活力增强，石泉、白河两县获得省政府“争先进位”表彰奖励。

城市建设力度加大。全面展开了中心城市总体规划修编和重点片区的详规工作，成功举办了安康城市发展战略论坛。汉江三桥、安康大道、花园大道、香溪大道等一批项目建成投用，江北新区基础建设和江南城区改造项目有序实施，南环干道、瀛湖干道、枣园路改造等项目相继开工，全市城镇基础设施建设完成投资8.8亿元。县城、集镇和村庄建设整体推进。在全市人民的大力支持和积极参与下，创建省级卫生城市和中国优秀旅游城市工作扎实展开，城市的卫生状况、交通秩序、市容市貌、文明程度有了明显改善和提升。汉阴、宁陕县通过省级卫生县城验收。汉阴县城被命名为省级园林县城。“双创”工作不仅改变了城市面貌，也促进了良好的工作机制、工作作风和文明习惯的形成。

生态旅游扎实推进。全市生态旅游发展总体规划颁布实施，秦巴风情、汉水神韵、金州美食、绿色安康的品牌开始叫响，“一山一湖一城”核心景区加快建设。我市与延长集团、陕旅集团联手实施大南宫山景区建设，标志着核心景区步入开发建设的新阶段。宁陕、石泉、汉滨、平利四大旅游板块各具特色，竞相发展。成功举办了旅游推介系列促销活动。南宫山景区被国际旅游联合会命名为中国最佳绿色生态景区，化龙山被国务院批准为国家级自然保护区，宁陕县通过省旅游强县验收，安康旅游知名度提升。全年接待游客300万人次，综合收入11亿元。

改善民生取得实效。全市财政投入民生支出23.7亿元，增长119%。列入规划的4055个民生八大工程项目全面完成，年初市政府承诺的十件实事如期实现。对当年未升学的25802名初高中毕业生实行了免费职业技能培训；新建和改扩建学校用房48万平方米；新增城镇就业1.3万人；新农合人均补助标准由40元提高到80元；城镇非从业人员基本医疗保险启动实施，对参保人员人均补助80元；建设农村敬老院197所、集中安置点358处，五保户集中供养率提高到50%；解决了6597户城镇困难户住房和3.1万贫困农民安居问题，廉租房建设获得省政府表彰；新修通乡油路400公里，通村水泥路2707公里，40万农民的出行难问题得到解决；西坝防洪保安工程开工建设；解决了16万农民的饮水安全，实现了11.2万贫困人口脱贫。

社会建设全面进步。教育强县区、强乡镇创建工作深入开展，免费义务教育政策落实到位，石泉县留守儿童教育经验在全国推广。旬阳“双高普九”县通过省政府验收。高考二本上线4893人，增长20%。基础教育、职业教育、高等教育和特殊教育全面发展。农村三级卫生网络和城市社区卫生服务体系逐步健全，新型农村合作医疗制度不断完善，妇幼保健和地方病防治得到加强。人口和计划生育工作水平有新的提高。新闻宣传发挥了正确的舆论引导作用，群众性的精神文明创建活动和全民健身运动多姿多彩。文化文物工作加强，文物普查扎实开展，文庙大成殿、北五省会馆等重点文物维修保护工程开工实施，首届政府文艺精品创作奖评选揭晓。信访积案集中办结和市县区领导大接访活动成效明显。平安安康创建活动不断深化，政法系统的基层基础建设全面推进，社会治安综合治理深入开展，公众安全感满意率位居全省第二。汉江干流水库防汛错峰调度和抢险救灾快速反应机制不断完善。安全生产形势平稳。残联、慈善协会、红十字会扶弱济困的作用愈加突出。各条战线、各个方面在发展与和谐的大局中都作出了积极贡献。

政府建设不断加强。深入推进思想解放，放眼安康发展面临的深刻变化，沉下去调查研究，走出去开阔视野，整体谋划安康突破发展，提出并实施了一系列新理念、新思路、新规划，使发展目标更加明确，工作思路更加清晰，推进措施更加有力。完善民主科学决策机制，自觉接受人大依法监督，密切与政协的民主协商，虚心接受人民群众的批评和建议，加强政风行风建设、行政监察和审计监督。抓住工作重点和薄弱环节，突出项目建设、招商引资和“双创”工作；认真落实应对金融危机、扩大内需的决策部署，及时编制项目规划，赴省进京汇报争取，一批项目列入中省计划，56个项目集中开工；出台了扩内需保增长的十条措施，有效解决企业面临的各种困难。扎实组织开展抗震救灾，全力筛查救治食用有害奶粉的婴幼儿，尽职尽责地保护人民群众健康和安全，忠实履行了为人民服务的崇高使命。

各位代表，过去的一年，我们不仅实现了经济社会发展的既定目标，同时对安康未来的突破发展做了进一步谋划。在思想大解放推动下，富民强市的发展目标进一步明确，加快新型工业化、城镇化、农业现代化和人文化进程的发展之路进一步明晰，产业兴市、项目第一的发展理念进一步确立，城乡统筹、经济社会协调发展的工作重点进一步突出，对外开放、招商引资的工作措施进一步强化，全市上下抓发展、促和谐的合力进一步凝聚。这是我们在深化市情认识、把握经济规律、推进突破发展的实践中取得的重要收获。

我们所取得的一切成绩，归功于科学发展观的正确指引，归功于全市人民的团结奋斗。在此，我代表市人民政府，向全体人大代表、政协委员，向奋战在各行各业、各条战线上的工人、农民、知识分子和干部职工，向各民主党派、工商联、人民团体、各界人士和离退休老同志，向驻安人民解放军、武警官兵、政法干警和复转军人，向所有关心支持安康建设的各界朋友，表示崇高的敬意和衷心的感谢！

我们的工作还存在不少的困难和问题。加快发展的基础条件还没有从根本上改变，经济结构调整的任务十分艰巨，新型工业化、城镇化、农业现代化建设相当紧迫，支撑全局发展的支柱产业、骨干企业和重大项目仍然缺乏，缩小与全省发展差距还要付出艰辛的努力。县域经济实力不强，工业基础尤为薄弱，财政收支矛盾突出，城乡居民收入偏低，就业压力日益增大。各类社会矛盾亟待化解，维护社会稳定的任务加重。投资环境不如人意，社会诚信需要提高，一些政府工作人员观念落后、作风拖沓，能力、素质和服务不适应发展的要求，落实工作还要进一步加强。我们必须正视存在的问题，采取更加有力的措施，在今后的工作中切实改进和努力解决。

二、当前形势和今年工作的总体要求

　 2009年是充满机遇、极具挑战的一年。国际金融动荡有可能持续较长时间，世界经济将经历一个较长的低迷和调整时期。我国经济下行风险比预想的要严重。受金融危机的波及，我市有色金属、矿产冶炼和缫丝企业效益下滑，主要农产品价格回落，农民工返乡增多，经济运行困难加剧。多年来经济社会生活中的矛盾和当前金融危机带来的影响相互交织，保增长、保稳定的社会环境趋紧。但是，我们要更加清醒地认识到，挑战中蕴含巨大的机遇，机遇大于挑战。国家实施积极的财政政策和适度宽松的货币政策，出台了扩大内需的一系列政策措施，大幅度增加基础设施、社会事业、改善民生、振兴产业的投入，宏观经济政策对我市的发展极为有利；国家和省上重点加强铁路、高速公路、机场和生态环境建设，安康是全省基础设施投资力度最大的区域之一；省委、省政府大力支持安康突破发展，支持在月河川道建设统筹城乡一体化发展实验区，支持“一体两翼”产业经济带和新材料基地建设，谋划的一批重大工业项目逐步开工实施，将显著增强经济实力和发展后劲；十县区全部纳入南水北调生态专项补偿范围，为进一步加强绿色生态环境建设提供了保障；东部产业和资本向中西部转移，为招商引资提供了巨大空间；西康高速和襄渝铁路复线今年通车，十天高速、川陕界高速加快建设，安康将融入西安两小时经济圈，真正形成沟通南北、连接东西的区域交通枢纽，大开放、大开发、大发展的时期正在到来。只要我们在困难中坚定信心、在挑战中把握机遇，就一定能够把不利影响降到最低，把各种机遇转化为新优势，实现经济社会平稳较快发展。

今年政府工作的总体要求是：高举中国特色社会主义伟大旗帜，深入贯彻落实科学发展观，围绕第一任务保增长、第一责任促稳定的要求，坚持以改革开放为动力，以项目建设为支撑，以招商引资为抓手，以改善民生为目的，着力夯实三大基础建设，做大做强六大主导产业，扎实抓好民生八大工程，加快“一体两翼”规划实施，促进县域经济快速发展，推进新型工业化、城镇化、农业现代化和人文化进程，努力建设生态、富裕、文明、和谐的新安康。

今年的主要发展指标建议为：生产总值270亿元，增长13%；全社会固定资产投资260亿元，增长30%；财政总收入30亿元，增长15%，其中一般预算收入9亿元，增长20%；全社会消费品零售总额95亿元，增长22%；城镇居民人均可支配收入11650元，净增1500元，农民人均纯收入3270元，净增500元；居民消费价格总水平涨幅控制在4.5%以下；城镇登记失业率控制在4.5%以内；人口自然增长率控制在4.5‰以内；单位生产总值能耗降低4.5%。

　在今年的经济社会发展中，必须把握好六项指导原则：

　把解放思想作为行为先导。准确把握安康发展环境的深刻变化，以更加开放的视野和胸襟谋划发展，以改革创新推进各项工作。

　把确保增长作为首要任务。紧紧抓住安康面临的发展机遇，依靠经济增长化解金融危机的影响，以增长的实绩解决发展中的困难和问题。

　把和谐稳定作为基本要求。切实落实保稳定第一责任，坚持以人为本，坚持依法治市，及时化解各类矛盾，着力营造有序的发展环境。

　把扩大投资作为工作重点。进一步加强项目建设和招商引资工作，突出新型工业的发展，保持固定资产投资的高位增长，加速提高工业在经济总量中的份额。

把改善民生作为为政之要。紧紧围绕群众增收，夯实各项民生基础，集中力量办好事办实事，在共享发展成果的基础上形成加快发展的巨大力量。

　把转变作风作为重要保证。适应发展环境的新变化和科学发展的新要求，在实施突破发展的实践中，使我们的工作方法、投资环境和落实工作有新的改进。

　 三、全力确保经济平稳较快增长

　保增长是今年政府工作的首要任务。实现保增长的既定目标，要着重抓好六个方面的工作。

　（一）以项目建设为支撑，确保投资和消费快速增长。抓住国家扩大内需增加投资的机遇，跑部进省加大项目争取力度，全力支持在建和规划的铁路建设、高速公路、机场迁建、汉江开发、生态保护等重大项目建设。加快陕川界、十天高速安康段的建设进度；确保西康高速公路5月底全线通车，武汉至安康至重庆铁路复线年内建成；开工建设西康铁路二线、西成铁路客运专线宁陕段、安康机场迁建、安康至平利关垭高速公路；争取列入规划的安康至张家界铁路、阳安铁路复线、陕渝高速安康至岚皋至镇坪、石泉至大河坝高速公路提前实施。力争宁陕筒车湾至镇安高速公路和西康高速旬阳连接线尽快开工。进一步加强城乡电网改造。着力抓好基础设施、绿色产业、民生工程和生态环境100个重点项目、60个前期项目,保持地方投资高位快速增长。把产业项目放在更加突出的位置，大幅提高产业投资、工业投资在固定资产投资中的比重。加快旬阳电站、蜀河电站、旬阳铁矿采选、汉滨五氧化二钒、50万吨铅锌选矿、南江河梯级电站、旬阳烟厂技改、大南宫山景区、宁陕朝阳沟景区、石泉汉江明珠景区10个项目建设进度；推进石泉火电厂、白河水电站、10万吨锌冶炼、陕重汽特种车改装、石泉激素类原料药开发、旬阳氯化钡、紫阳锆钢玉、岚皋复合脱氧剂、宁陕钼矿采选、白河硫酸10个项目开工；力促蜀河电站首台机组、阳晨20万吨饲料、平利硅钡钙、江华水泥、金龙水泥、秦东魔芋胶囊、旬阳铜矿采选、镇坪和平利腊肉精加工、石泉鎏金蚕丝绢、岚皋煤矸石增碳剂10个项目竣工投产；加大多晶硅、硫酸镍、金属镁及镁合金、海绵钛、锌合金、海绵铁、汽车球磨铸件、汉阴页岩开发、旬阳火电、燃料乙醇10个重大项目前期工作力度。下决心改变工业项目缺乏、重大项目薄弱的状况，市县区财政从今年起每年安排5000万元项目前期经费，用于重大项目的策划、包装、可研、论证和招商，聘请高等院校、专业机构、科研单位领衔重大项目的前期工作。全面落实刺激消费的各项措施，努力增加城乡居民收入，引导消费观念转变，积极发展商贸、流通、餐饮等服务业，鼓励旅游休闲、卫生保健、文化娱乐、汽车等新兴消费。努力增加政府公共支出，加大民生项目和市政建设的投入，开工建设市本级30万平方米经济适用房、县区经济适用房小区和10万平方米廉租房建设，用足用活住房公积金贷款和廉租房补贴政策。积极发展节庆、会展、假日经济，精心组织汉江龙舟节、茶文化节和油菜花节，迎接西康高速通车人流物流高峰，充分发挥拉动消费、招商引资、休闲旅游、开放交流的作用。努力扩大农村消费市场，继续落实“家电下乡”补贴政策，推进“万村千乡”市场工程，启动县城中型超市建设，促进消费结构升级和人民生活水平提高。

　（二）以新型工业化为引擎，助推经济提速增效。坚持大集团引领、大项目推动、集群化发展、园区化承载，做大做强工业支柱产业，全年工业产值增长20%以上，新增规模企业25户。发挥规划的引领作用，抓紧评审报批和启动实施“一体两翼”区域发展规划和新型材料工业规划，在做好矿产选冶的基础上，围绕新型金属材料、无机非金属材料、硅材料、新型建筑材料四大材料体系，包装成形项目，合理规划布局，抓紧招商落地，尽快形成工业增长点。使“一体两翼”成为要素集中、产业聚集、生态环保、带动全局的产业经济带，把新材料工业基地建成技术先进、结构优化、链条完备、产业均衡的循环经济示范区。以密切与大企业合作为重点，加快汉江干流三座大型电站和石泉、旬阳两座大型火电厂建设，加大原料基地建设力度，促进生物柴油和燃料乙醇项目的实施，毫不放松核电项目前期工作，壮大清洁能源工业。加快以富硒为特色的食品加工工业发展，毫不放松生物医药和丝绸产业的振兴。提高安康工业园区建设水平，加快完善体制、机制和政策，引导工业企业和新上项目向园区集中，提升园区的服务、承载和招商能力。认真落实工业经济保产能、保增长的各项政策措施，全力支持有市场、有效益的工业企业开足马力生产，努力促进拉动内需的企业和产品扩大规模，切实帮助有色金属、矿产冶炼和缫丝行业走出低谷，充分利用原材料和装备价格下滑的有利时机建设工业项目，帮助支持小企业在发展中成为规模企业，鼓励引导各类市场主体向工业领域迈进。强化特色品牌建设和名牌产品培育，增强企业核心竞争力。加强对骨干企业、重点产业的政策扶持和政府服务，切实落实减税让利、清理收费、财政贴息、财信担保、金融支持等各项措施，力保工业经济在应对金融危机中较快增长。尊重企业家的创造，加强企业家队伍建设，保护好企业发展的积极性，在全社会形成竞相创办企业和重视企业家、支持企业家、保护企业家的良好氛围。

　 （三）以农民增收为中心，努力壮大县域经济实力。认真贯彻落实党的十七届三中全会和今年中央一号文件精神，推进农民增收、农业发展、农村繁荣。坚定不移地走“一县一业、一村一品、强村大户、龙头牵引”的县域经济发展路子，统筹产业化、工业化、城镇化发展。围绕稳定80万吨粮食，建设200万亩桑、茶、烟、果、药、菜等特色种养业基地，发展500万头商品猪，开发30万亩水产养殖，输出60万劳务的五年规划目标，大力发展规模、特色、效益农业，确保今年农业增长6%。加快土地经营权流转，全面推开林权制度改革，积极启动户籍制度改革，转变农业发展方式，催生农业规模经营。增加农村基础设施建设投入，新修基本农田5万亩、沼气2万口。全面实施农业综合开发，整体推进扶贫攻坚，启动462个新农村示范村建设。完善农产品市场和农村商业网点体系，加快南水北调水保治理和长治水保项目的实施，全面启动汉江沿岸绿化造林工程。巩固扩大劳务市场，稳定50万人输出规模，提高组织化程度和工资性收入。高度重视和抓好返乡农民工的就业培训，引导他们围绕项目实施、工业发展和新农村建设就地转移。加快城镇化进程，加强规划指导和实施，完善县城和小城镇基础设施，建设沿汉江县城污水和垃圾处理项目，重点建设20个中心集镇。把新型工业作为县域经济发展的重点，启动旬阳、汉阴、石泉、紫阳四个县域工业园区建设，抓紧汉滨、平利等6个工业园区报批。把工业增长作为年度目标考核的重点内容。支持旬阳加快实施工业倍增计划，帮助汉滨不断催生新的产业增长点，促进平利、汉阴、石泉实施率先突破，推动白河、宁陕、紫阳、岚皋、镇坪尽快培育壮大特色产业和骨干企业。深化扩权强县试点，鼓励县域经济争先进位，激励有实力的摘帽脱贫，形成各县区竞相发展的生动局面。

　 （四）以中心城市建设为引领，切实提升城镇化水平。紧扣建设山水园林城市和旅游目的地的定位，加快构建适应大交通、大旅游、大发展的中心城市、县城、小城镇体系。按照百万人口的发展规模，聘请国内一流专家，组织本市知名人士，发动市民积极参与，高质量完成中心城市总体规划修编。江北新区要展现现代城市风貌，江南改造要体现历史文化特色，汉江两岸要凸显独特魅力神韵。着眼于中心城市的长远发展和实际需要，总体规划和分年实施城东汉江大桥、广播电视中心、安康大剧院、体育运动中心、儿童医院、市民文化中心、博物馆、图书馆、汉滨行政中心、安康学院十大公共服务项目和会展中心、商贸步行街、高星级酒店、商务大厦、物流中心、旅游服务中心、国税大厦、电力大厦、交通大厦、信合大厦十大经营服务项目，把这些项目建成凝聚建筑精华、凸显文化特色、代表城市形象的标志性建筑。抓紧江北新区、汉江两岸、东西两坝等重点片区的详细规划，合理布局和建设特色鲜明、协调配套的功能区。促进首批北迁单位的规划、定点、设计、筹资和开工。推进东西坝防洪工程及片区改造、东关和新城片区改造、江北污水处理厂、滨江大道三期、城区天然气管网、市民广场、江北江南重点路段改建等50个项目，今年完成投资7亿元。高水平设计、高品位实施中心城市和城郊绿化、美化、亮化工程，打造城在绿中、人在林中，特色鲜明的城市景观带。促进房地产业持续健康发展，支持建筑业做大做强。牢固树立规划立市的理念，深化城市建设和管理体制改革，加快提升规划、建设和管理水平。严禁未批先建、违规擅建、乱批乱建，切实呵护好山、水、城相融通的城市整体形态。按照突出特色、完善功能、承载发展的要求，努力把各县城建设成为县域经济社会发展的中心。全力打好“双创”第二阶段攻坚战，深入开展14项专项整治，加大城市硬件设施投入，加快城区农贸市场、停车场、垃圾中转站、餐饮场所、公厕等配套设施建设。加大铁路、高速公路、国省道沿线、城乡结合部的环境综合整治和特色民居建设。平利、白河实现省级卫生县城创建目标，紫阳达到市级卫生县城标准。在“双创”中一定要使广大市民文明素质提升、人居环境改善、出行安全方便、生活质量提高。

　 （五）以生态旅游为龙头，促进特色服务业快速成长。认真实施安康旅游总体规划，突出生态旅游品牌定位，着眼大旅游、大市场、大产业的发展，着力打造“一山一湖一城”核心景区。加快大南宫山景区道路建设，开工建设真身殿等重点工程。下功夫做好大瀛湖景区的规划包装，引进大企业集团整体开发建设，把瀛湖建设成为亲水休闲的“西部千岛湖”。加快构建宁陕森林体验、石泉融山亲水、平利田园风光、汉滨水乡休闲四大旅游板块。以道路和码头建设引导景区开发，推进天华山、朝阳沟、燕翔洞、中坝峡谷、天书峡、汉江两岸等重点景区建设。打造西安－宁陕秦岭森林体验一日游，西安－石泉－汉阴－紫阳－安康汉水风光两日游，安康－岚皋－平利巴山风情三日游精品线路。加强与省内外的旅游合作交流，把安康融入旅游大格局之中，建设国内闻名的生态旅游胜地，打造西北美丽的乡村旅游示范区。迎接西康高速通车后的旅游高峰，成立旅游指挥中心和游客服务中心，落实吃、住、行、游、购、娱应对方案，加快改造宾馆、饭店、停车场等服务设施，大力提升城镇周围、景区周边和旅游通道沿线的承载能力，全年接待游客500万人次，综合收入20亿元。大力发展交通运输、宾馆饭店、文化休闲、特色市场等服务业，加强物流园区、市场体系的规划建设，培育一批大型超市、保鲜冷藏、包装储运、专业批发、物流配送等骨干商流企业，努力把安康建设成为特色鲜明、安全快捷的区域物流中心。

　 （六）以改革开放为动力，推动安康加快突破发展。开放经济迫切要求我们深入解放思想，大胆改革创新，跳出安康发展安康，主动融入西安、成渝和江汉经济圈，在大市场中谋求新的发展机遇。紧紧扭住招商引资不放松，围绕优势资源、新型工业、城市建设、生态旅游、现代农业、交通物流等策划推出一批项目，采取领导招商、叩门招商、以企招商、以商招商、中介招商、网络招商等有效形式，瞄准省内外大企业，特别是我省延长、有色、陕重汽、陕医药等企业集团，实施重点突破。有效承接东部产业转移，积极实施走出去战略，建立发达地区定点招商平台，今年集中到广东、浙江等地举行4次以上招商推介活动；各县区都要走出去、请进来，寻求更多的招商机遇。实行招商引资县区和部门一把手负责制，严格目标责任考评，确保全年招商引资到位资金35亿元。推行招商引资部门联审制，把审批环节减少到最低程度。建立落地重点项目全程服务制，及时解决项目建设中的具体问题。按照建设省级新型工业、高效农业、特色服务业、现代城镇体系综合实验区的要求，抓紧编制月河川道统筹城乡一体化发展实验区规划，组织强有力的工作班子，完善实验区建设机制，争取优惠电价、土地审批、项目核准等特殊产业政策，分年度、有步骤强力推进。认真落实促进民营经济和全民创业的政策措施，加强创业培训、指导和服务，支持各类市场主体、大中专毕业生、复转军人、下岗职工、外出务工人员、农村能人大户创办企业，掀起全民创业热潮。加大资本市场运作力度，鼓励有条件的企业上市融资；按照现代企业制度要求，抓紧完善城投公司治理结构和运行机制，建立市政建设投融资平台；充分运用政府信用，募集集合信托资金，拓宽投融资渠道；进一步扩大财信担保公司资本金，增强服务中小企业的能力；深化与国家开发银行的合作，推进市内银行创新地方建设融资产品；鼓励其他商业银行在安康建立分支机构，积极争取国际金融组织和外国政府贷款；创造条件开展小额贷款公司试点工作，拓展全民创业的融资渠道；建立财政贴息制度，拿出3000万元贴息资金，支持企业的正常生产经营。积极争取省上的特殊政策，改进项目审批、用地、环评等工作。加强诚信安康建设，下茬改善投资环境。开展优化投资环境年活动，狠刹办事拖拉、故意刁难、吃拿卡要、雁过拔毛等歪风邪气，在全社会营造人人都是投资环境、人人关心安康发展、人人竞相多做贡献的良好风气。要让那些破坏安康投资环境的人没有立足之地、让那些损害安康发展形象的人付出沉痛代价。

　四、努力促进社会和谐稳定发展

　 社会和谐是保增长促发展的前提和基础，我们必须统筹经济社会发展，更好地关注民生，更多地增加社会事业投入，更有力地推进和谐社会建设。

　 深入实施民生八大工程。按照规划确定的年度目标，密切与省上沟通衔接，加大项目争取力度，足额落实配套资金，完成八大类4000个项目、投资25亿元的年度计划。在整体推进的基础上，市政府重点抓好十件实事。①完善以就业为中心的职业教育体系，对未升学的初高中毕业生一个不少的实行免费技能培训，为1.3万新增劳动力和下岗职工提供就业岗位。②加强城乡卫生服务体系建设，进一步完善农村新合疗制度，扩大城镇居民基本医疗保险覆盖面。③增强城乡困难人口的住房保障能力，解决9626户城镇特困户的住房和农村3万贫困人口的安居问题。④提高城乡居民最低生活保障水平，城市低保人均月补助提高30元，农村年低保标准再增加92元。⑤进一步抓好农村养老工程，新改扩建农村敬老院25所，五保户集中供养率达到60%，每人年供养补助标准再提高216元。启动新型农村社会养老保险试点。⑥继续推进农村公路建设，新修通乡油路400公里，通村水泥路2000公里，实现90%的村通水泥路。⑦加快农村安全饮水工程建设，新建饮水工程300处，让18万农民用上清洁安全的自来水。⑧加大扶贫攻坚工作力度，启动139个移民扶贫示范村、150个扶贫重点村、130个扶助资金试点村建设，减少贫困人口8万人。⑨实施农村信息化工程，实现1500个20户以上的自然村广播电视“村村通”，60%的行政村接入互联网。⑩开工东坝防洪保安工程，加快西坝防洪工程建设，根治危及东西两坝的水患之害。

　推动社会事业协调发展。把教育放在优先发展的位置。巩固城乡义务教育成果，完善义务教育经费保障机制，加大对教育事业的投入。适应城市发展和人民群众对教育事业的新要求，整合和优化配置中心城市教育资源，发挥名校的辐射带动作用，加快陈家沟小学和果园小学建设进度，扩大安康中学、汉滨高中办学规模，缓解城区教育资源短缺状况。深入开展教育强县区、强乡镇创建工作，旬阳、石泉实现省级教育强县建设目标，宁陕“双高普九”取得阶段性成果。加快首批14所农村寄宿制学校建设。努力扩大高中教育办学规模，支持特殊教育、幼儿教育和民办教育发展，办好高等教育和职业技术教育，启动安康学院、安康职业技术学院和安康技术学院北迁工程。深入实施科技兴安战略，完善自主创新激励机制，加大科技成果转化和先进技术推广力度，加强与西安高新区、杨凌农业示范区的合作，密切与高等院校、科研院所、企业集团的技术交流，提升我市的产业层次、发展方式、产品结构和创新能力。继续加强城乡公共卫生体系建设，抓好重大疾病预防控制和地方病防治，提高应对突发公共卫生事件的能力。推进市人民医院医技大楼、中医院综合楼建设，支持办好县医院、乡镇卫生院和村卫生室，加强社区卫生工作。提高儿童免疫率和孕产妇住院分娩率。坚持以稳定低生育水平为目标，强化基层基础工作，严格执行“一票否决”和一把手负责制，落实农村计生家庭奖扶政策，逐步开展乡镇卫生院和计划生育服务站资源整合，从今年开始，连续三年每年从乡镇现有卫生、计生专业人员中选送200名到市职业技术学院进行培训。加快公共文化体系建设，完善政府文艺精品奖评奖办法，加强汉水文化研究和文化遗产保护，全面完成第三次文物普查任务，推动文化产业与生态旅游融合互动。加强精神文明建设，推进新闻、出版、广播、电视、信息事业发展，促进文化大发展大繁荣。广泛开展全民健身运动，办好全市第十四届运动会。加快少数民族聚集区经济社会发展，尽快改善少数民族群众的生产生活条件。

　 保持社会和谐稳定局面。充分认识现阶段各类社会矛盾多发易发的现实，切实增强保增长第一要务、保稳定第一责任的意识，从基层基础工作抓起，从理顺群众情绪入手，妥善处理各类社会矛盾，完善维护社会稳定的工作机制。进一步加强信访工作，变群众上访为干部下访，抓紧信访积案办理。深化“平安安康”创建，加强社会治安综合治理，依法打击各类犯罪，增强广大人民群众的安全感。整合基层信访、公安、司法、行政复议、人民调解等各种机制和力量，抓早抓小，把矛盾和问题最大限度解决在基层。夯实安全生产责任，严防重特大事故发生。强化市场监管，确保食品药品安全。始终把防汛减灾摆在突出位置，加强汉江防汛和山地灾害防范。完善应急管理机制，提高应对自然灾害、事故灾难、公共卫生和社会安全事件的能力。统筹人与自然的和谐发展，深入开展节能减排全民行动，大力倡导节约、环保、文明的生产和消费方式，努力完成节能降耗减排任务，保持汉江安康段Ⅱ类水质。积极发展残疾人、慈善和老龄事业，不断完善政府保障、社会援助制度。依法加强宗教事务管理。严密防范各类邪教对人民群众精神健康和社会安定的危害。重视做好对台工作。加强国防后备力量建设，深入开展双拥共建。支持工会、共青团、妇联、工商联等人民团体在和谐社会建设中更好地发挥作用。

　 五、进一步加强政府建设

　适应新的形势，顺应人民期待，不负时代重托，是全市人民对政府工作的期望和要求。我们要在科学发展观的指导下，坚持科学行政、依法行政，恪尽职守，不辱使命。

　 坚持依法行政，建设法治政府。认真贯彻依法治市方略，把依法行政贯穿于政府决策、执行、监督的全过程，建立权责明确、行为规范、监督有效、保障有力的行政执法体系，严格按照法定权限和程序行使权力、履行职责、维护公平正义。依法管理经济社会事务，以法治政府建设带动法治社会建设。教育引导各类组织、全体公民依法行为，自觉维护法律法规尊严，增强尊重国家、集体及其他公民合法权益的社会意识，使一切经济社会活动都在法律轨道上健康运行。对置法律于不顾、公然挑衅社会正义的极端个人主义，必须依法坚决制止；对肆意践踏法律、严重侵害公众利益和公共秩序的违法行为，必须依法予以打击。认真贯彻落实省委、省政府和市委的决策部署，自觉接受市人大及其常委会的依法监督，支持政协履行政治协商、民主监督和参政议政职能，主动听取各民主党派、工商联、无党派人士和人民团体的意见，接受新闻舆论、社会公众和政府系统内部监督，支持监察、审计等部门依法独立履行监督职责。

　坚持科学行政，建设效能政府。进一步完善决策机制，坚持科学民主决策。对涉及安康经济社会发展的重大事项，要充分进行评估论证，主动提请人大依法审议、政协民主协商、党委研究决定；关系群众切身利益的重大问题实行公开听证、新闻发布和决策公示制。努力加强学习，建设学习型机关，提高指导经济工作的专业水平。切实转变作风，提高工作效率。大力倡导深入实际、雷厉风行，坚决防止官僚主义、形式主义；可开可不开的会坚决不开，可发可不发的文件一律不发，提倡开短会、现场会、电视电话会和少发文、发短文，提高实效性和针对性。完善目标责任制，提高执行力、公信力，实行问责制，健全抓落实机制，加强政府督查，限时督办和效能监察，确保政令畅通和高效运转。

　坚持执政为民，建设服务政府。扎实开展科学发展观学习实践活动，把学习实践活动与安康突破发展、思想观念转变、解决实际问题、富裕全市人民紧密结合，达到干部受教育、发展上水平、群众得实惠的目的。深怀爱民之心，恪守为民之责，善谋富民之事，把执政为民落实在具体行动上，真心实意地服务基层、服务企业、服务人民。不断创新政府管理理念、管理机制、管理手段，精心组织市县区政府机构改革，实行大部门制，形成权责一致、分工合理、执行顺畅、监督有力的行政管理体制，更好地履行经济调节、市场监管、公共服务和社会管理职能。

　坚持从严治政，建设廉洁政府。全面贯彻落实标本兼治、综合治理、惩防并举、注重预防的方针带头执行廉洁自律各项规定，自觉履行廉政建设责任制，严肃惩处各种腐败行为。严格要求、严格教育、严格管理、严格监督政府工作人员，努力形成靠制度管人治权理事的机制。深入推进政务公开，深化政风行风评议工作，保障人民群众的知情权、参与权、表达权和监督权。始终保持不骄不躁、艰苦奋斗的作风，厉行勤俭节约，珍惜民力民财，狠刹奢侈浪费。今年公车购置、会议经费、公务接待和公款出国等经费支出实行零增长，新增财力要向保增长倾斜，向民生倾斜，向基础建设倾斜，切实降低行政成本，树立人民政府的良好形象。

　各位代表！今年是安康发展史上极为关键、十分重要的一年。随着西康高速的正式通车，安康将站在大开放的新起点上。能否抓住机遇，迎难而上，加快发展，是对我们信心、决心、胆略和智慧的考验。我坚信，任何困难都改变不了安康人民创造美好生活的执着追求，任何挑战都阻挡不了安康人民加快突破发展的坚定步伐。让我们高举科学发展观的伟大旗帜，在省委、省政府和市委的坚强领导下，团结一心，锐意进取，共克时艰，以突破发展的新成绩，迎接新中国成立六十周年！
